# Periodontitis and Outer Retinal Thickness: a Cross-Sectional Analysis of the United Kingdom Biobank Cohort

**DOI:** 10.1016/j.xops.2024.100472

**Published:** 2024-01-20

**Authors:** Siegfried K. Wagner, Praveen J. Patel, Josef Huemer, Hagar Khalid, Kelsey V. Stuart, Colin J. Chu, Dominic J. Williamson, Robbert R. Struyven, David Romero-Bascones, Paul J. Foster, Anthony P. Khawaja, Axel Petzold, Konstantinos Balaskas, Mario Cortina-Borja, Iain Chapple, Thomas Dietrich, Jugnoo S. Rahi, Alastair K. Denniston, Pearse A. Keane, UK Biobank Eye & Vision Consortium, Naomi Allen, Tariq Aslam, Denize Atan, Konsantinos Balaskas, Sarah A. Barman, Jenny H. Barrett, Paul Bishop, Graeme Black, Tasanee Braithwaite, Roxana O. Carare, Usha Chakravarthy, Michelle Chan, Sharon Y.L. Chua, Alexander Day, Parul Desai, Bal Dhillon, Andrew D. Dick, Alexander Doney, Cathy Egan, Sarah Ennis, Paul Foster, Marcus Fruttiger, John E.J. Gallacher, David F. Garway-Heath, Jane Gibson, Jeremy A. Guggenheim, Chris J. Hammond, Alison Hardcastle, Simon P. Harding, Ruth E. Hogg, Pirro Hysi, Pearse A. Keane, Sir Peng T. Khaw, Anthony P. Khawaja, Gerassimos Lascaratos, Thoams Littlejohns, Andrew J. Lotery, Robert Luben, Phil Luthert, Tom Macgillivray, Sarah Mackie, Bernadette McGuinness, Gareth J. McKay, Martin McKibbin, Tony Moore, James E. Morgan, Eoin O’Sullivan, Richard Oram, Chris G. Owen, Praveen Patel, Euan Paterson, Tunde Peto, Axel Petzold, Jugnoo S. Rahi, Alicja R. Rudnikca, Naveed Sattar, Jay Self, Panagiotis Sergouniotis, Sobha Sivaprasad, David Steel, Irene Stratton, Nicholas Strouthidis, Cathie Sudlow, Zihan Sun, Robyn Tapp, Dhanes Thomas, Emanuele Trucco, Adnan Tufail, Veronique Vitart, Ananth C. Viswanathan, Mike Weedon, Cathy Williams, Katie Williams, Jayne V. Woodside, Max M. Yates, Jennifer Yip, Yalin Zheng

**Affiliations:** 15 University of Oxford; 16 University of Manchester; 17 University of Bristol; 18 Moorfields Eye Hospital; 19 Kingston University; 20 University of Leeds; 21 Manchester University; 22 University of Manchester; 23 St Thomas’ Hospital; 24 University of Southampton; 25 Queens University Belfast; 26 NIHR Biomedical Research Centre; 27 NIHR Biomedical Research Centre; 28 NIHR Biomedical Research Centre; 29 NIHR Biomedical Research Centre; 30 University of Edinburgh; 31 University of Bristol; 32 University of Dundee; 33 NIHR Biomedical Research Centre; 34 University of Southampton; 35 NIHR Biomedical Research Centre; 36 NIHR Biomedical Research Centre; 37 University of Oxford; 38 NIHR Biomedical Research Centre; 39 University of Southampton; 40 Cardiff University; 41 King’s College London; 42 NIHR Biomedical Research Centre; 43 University of Liverpool; 44 Queens University Belfast; 45 King’s College London; 46 NIHR Biomedical Research Centre; 47 NIHR Biomedical Research Centre; 48 NIHR Biomedical Research Centre; 49 NIHR Biomedical Research Centre; 50 University of Oxford; 51 University of Southampton; 52 UCL; 53 UCL; 54 University of Edinburgh; 55 University of Leeds; 56 Queen’s University Belfast; 57 Queen’s University Belfast; 58 Leeds Teaching Hospitals NHS Trust; 59 NIHR Biomedical Research Centre; 60 Cardiff University; 61 King’s College Hospital NHS Foundation Trust; 62 University of Exeter; 63 University of London; 64 NIHR Biomedical Research Centre; 65 Queens University Belfast; 66 Queen’s University Belfast; 67 UCL; 68 UCL Great Ormond Street Institute of Child Health; 69 University of London; 70 University of Glasgow; 71 University of Southampton; 72 University of Manchester; 73 NIHR Biomedical Research Centre; 74 Newcastle University; 75 Gloucestershire Hospitals NHS Foundation Trust; 76 NIHR Biomedical Research Centre; 77 University of Edinburgh; 78 UCL; 79 St George’s University of London; 80 NIHR Biomedical Research Centre; 81 University of Dundee; 82 NIHR Biomedical Research Centre; 83 University of Edinburgh; 84 NIHR Biomedical Research Centre; 85 University of Exeter; 86 University of Bristol; 87 King’s College London; 88 Queen’s University Belfast; 89 University of East Anglia; 90 University of Cambridge; 91 University of Liverpool; 1 Population and Data Sciences, Institute of Ophthalmology, University College London, London, United Kingdom; 2 NIHR Biomedical Research Centre, Moorfields Eye Hospital and UCL Institute of Ophthalmology, London, United Kingdom; 3 Department of Ophthalmology and Optometry, Kepler University Hospital, Linz, Austria; 4 Centre for Medical Image Computing, Department of Computer Science, University College London, United Kingdom; 5 Biomedical Engineering Department, Faculty of Engineering (MU-ENG), Mondragon Unibertsitatea, Mondragón, Spain; 6 Department of Neuroinflammation, Queen Square Institute of Neurology, University College London, London, United Kingdom; 7 Population, Policy and Practice, Great Ormond Street Institute of Child Health, University College London, London, United Kingdom; 8 NIHR Birmingham Biomedical Research Centre, University of Birmingham, Birmingham, United Kingdom; 9 Institute of Inflammation and Ageing, University of Birmingham, Birmingham, United Kingdom; 10 School of Dentistry, Birmingham Community Healthcare NHS Foundation Trust, United Kingdom; 11 NIHR Biomedical Research Centre at UCL Great Ormond Street Institute of Child Health and Great Ormond Street Hospital, London, United Kingdom; 12 Department of Ophthalmology, Great Ormond Street Hospital NHS Foundation Trust, London, United Kingdom; 13 Ulverscroft Vision Research Group, Institute of Child Health, University College London, London, United Kingdom; 14 Department of Ophthalmology, University Hospitals Birmingham NHS Foundation Trust, Birmingham, United Kingdom

**Keywords:** Periodontitis, Age-related macular degeneration, Optical coherence tomography

## Abstract

**Purpose:**

Periodontitis, a ubiquitous severe gum disease affecting the teeth and surrounding alveolar bone, can heighten systemic inflammation. We investigated the association between very severe periodontitis and early biomarkers of age-related macular degeneration (AMD), in individuals with no eye disease.

**Design:**

Cross-sectional analysis of the prospective community-based cohort United Kingdom (UK) Biobank.

**Participants:**

Sixty-seven thousand three hundred eleven UK residents aged 40 to 70 years recruited between 2006 and 2010 underwent retinal imaging.

**Methods:**

Macular-centered OCT images acquired at the baseline visit were segmented for retinal sublayer thicknesses. Very severe periodontitis was ascertained through a touchscreen questionnaire. Linear mixed effects regression modeled the association between very severe periodontitis and retinal sublayer thicknesses, adjusting for age, sex, ethnicity, socioeconomic status, alcohol consumption, smoking status, diabetes mellitus, hypertension, refractive error, and previous cataract surgery.

**Main Outcome Measures:**

Photoreceptor layer (PRL) and retinal pigment epithelium–Bruch’s membrane (RPE–BM) thicknesses.

**Results:**

Among 36 897 participants included in the analysis, 1571 (4.3%) reported very severe periodontitis. Affected individuals were older, lived in areas of greater socioeconomic deprivation, and were more likely to be hypertensive, diabetic, and current smokers (all *P* < 0.001). On average, those with very severe periodontitis were hyperopic (0.05 ± 2.27 diopters) while those unaffected were myopic (−0.29 ± 2.40 diopters, *P* < 0.001). Following adjusted analysis, very severe periodontitis was associated with thinner PRL (−0.55 μm, 95% confidence interval [CI], −0.97 to −0.12; *P* = 0.022) but there was no difference in RPE–BM thickness (0.00 μm, 95% CI, −0.12 to 0.13; *P* = 0.97). The association between PRL thickness and very severe periodontitis was modified by age (*P* < 0.001). Stratifying individuals by age, thinner PRL was seen among those aged 60 to 69 years with disease (−1.19 μm, 95% CI, −1.85 to −0.53; *P* < 0.001) but not among those aged < 60 years.

**Conclusions:**

Among those with no known eye disease, very severe periodontitis is statistically associated with a thinner PRL, consistent with incipient AMD. Optimizing oral hygiene may hold additional relevance for people at risk of degenerative retinal disease.

**Financial Disclosure(s):**

Proprietary or commercial disclosure may be found in the Footnotes and Disclosures at the end of this article.

Periodontal disease is a holistic term used to describe a group of common chronic disorders of the gums that are initiated by accumulation of a dental plaque biofilm on the teeth, but which are characterized by inflammation of the periodontal tissues, including the alveolar bone that surrounds the teeth. Typically, periodontal disease progresses from an early reversible form, termed gingivitis, where the gums may swell and bleed, to very severe periodontitis which is a major cause of tooth loss and gingival recession if left untreated.^1^^,^^2^ Up to half of adults worldwide are estimated to have irreversible periodontitis with a peak prevalence of severe disease in those aged 60 to 64 years.^3^, ^4^, ^5^ Periodontitis is independently associated with several chronic inflammatory noncommunicable diseases of aging, such as type 2 diabetes,^6^ atherogenic cardiovascular disease,^7^ and associated major adverse cardiovascular events,^8^ chronic kidney disease,^9^ rheumatoid arthritis,^10^ and Alzheimer’s disease.^11^ Biological mechanisms of association include periodontal bacteremia during daily function due to microulcers in the gingival (gum) lining, dissemination inflammation from the periodontal tissues, and posttranslational sequelae of periodontal inflammation that generate autoantigens within periodontal tissues and may predispose to systemic autoimmune disease.^10^

Given the role of chronic inflammation in the pathogenesis of age-related macular degeneration (AMD), several epidemiological investigations have sought to investigate the link between AMD and periodontal disease.^12^ Population-based health surveys in Finland, South Korea, and the United States have found an increased prevalence of AMD in individuals with periodontitis, particularly among those < 60 years of age,^13^, ^14^, ^15^ suggesting that severe periodontitis may contribute to the premature development of AMD. Supporting this hypothesis, an analysis of the National Health Insurance Research Database in Taiwan over a 12-year period found that individuals with periodontitis had 58% greater hazard of developing AMD compared with those without.^16^ However, the findings were based on routinely collected retrospective data where there is risk of residual confounding (e.g., smoking status was not included despite strong links with periodontitis and AMD) and use of diagnostic codes for the case definition may be prone to information bias. Moreover, the specific date of disease codes, such as AMD and periodontitis, which are asymptomatic at their early stages, may not be representative of actual disease development. A further limitation of all the above reports is that the diagnosis of AMD is based on color fundus photography (CFP), yet the detection of disease-related features, such as drusen and atrophy of the retinal pigment epithelium (RPE), are greater with OCT.^17^^,^^18^ Assessment of OCT-based sublayer thicknesses has increasingly recognized an association between thinning of the outer retinal layer and thickening of the retinal pigment epithelium–Bruch’s membrane (RPE–BM) layer in both early and incipient AMD.^19^, ^20^, ^21^

In this study, we explored the association between very severe periodontitis and outer retinal sublayers using deeply phenotyped data from the prospective community-based research cohort, United Kingdom (UK) Biobank (UKBB). Our objective was to investigate whether individuals with very severe periodontitis and no eye disease had outer retinal OCT features suggestive of early AMD. We hypothesized that affected individuals would have reduced thickness of the photoreceptor layer (PRL) and increased thickness of the RPE–BM.

## Methods

### Data and Design

We conducted a cross-sectional analysis of data from the UKBB, a prospective epidemiological cohort study of > 500 000 participants aged between 40 and 70 years and residing in the UK. Participants were recruited between 2006 and 2010 and gave informed consent to undergo deep phenotyping for the investigation of health and disease (more information available at: https://www.ukbiobank.ac.uk/). As part of a touchscreen questionnaire at their initial assessment visit, participants were asked about oral/dental problems experienced within the last year. A subset of 67 321 UKBB participants additionally underwent a detailed ophthalmic assessment including retinal imaging with both CFP and OCT at their initial assessment visit.^22^^,^^23^

Retinal imaging within UKBB was acquired using the Topcon 3D-OCT 1000 device (Topcon Corporation). All images covered a 6.0-mm^2^ × 6.0-mm^2^ area and had 128 horizontal B-scans and 512 A-scans per B-scan. Images from both eyes, where available, were used. Only participants who had completed the touchscreen questionnaire and undergone retinal imaging were included. Those who had retinal imaging only at the second assessment visit (2012–2013) were excluded, as this would be a significant duration from the recording of periodontitis. Those who self-reported any eye disease were also excluded, as this may interfere with the retinal imaging measures.

### Outcome Variables

**Figure 1:**
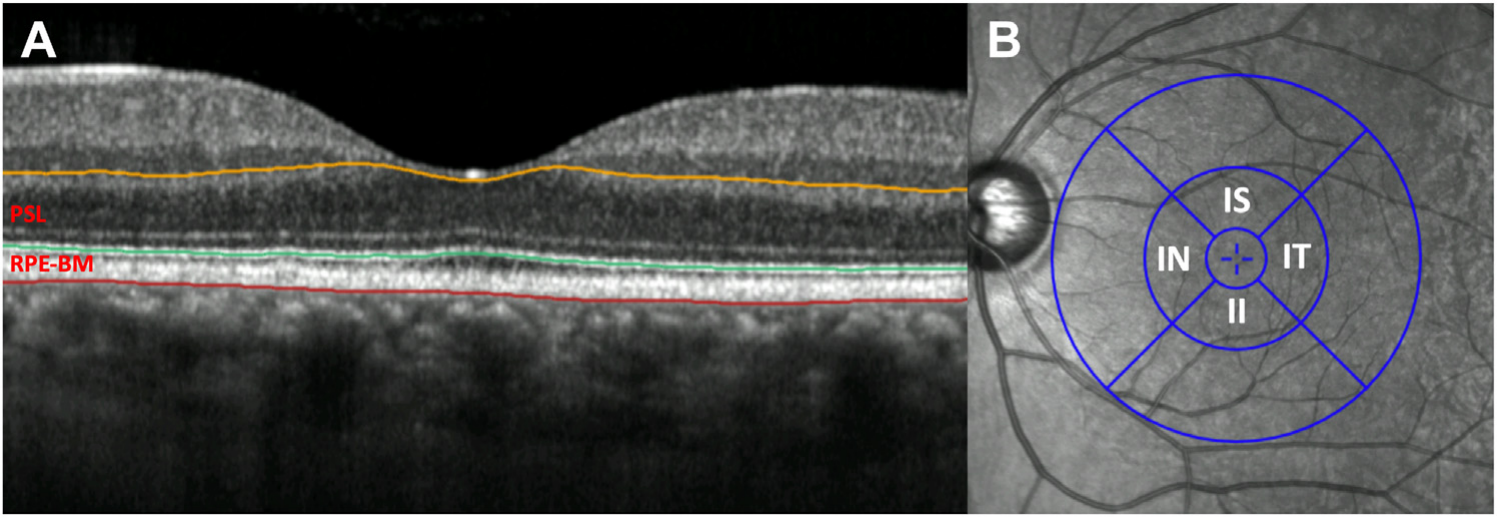
Example macular OCT B-scan showing segmented boundaries of the photoreceptor segment (orange to green) and retinal pigment epithelium–Bruch’s membrane (RPE–BM) (green to red) layers (**A**). Layer thicknesses were extracted from the parafoveal segments indicated (**B**). II = inner inferior; IN = inner nasal; IS = inner superior; IT = inner temporal.

The primary outcome measures were PRL and RPE–BM thickness, derived from automated segmentation of OCT. OCTs were segmented using the Topcon Advanced Boundary Segmentation tool (version 1.6.2.6), a software leveraging dual-scale gradient information for automated segmentation of retinal sublayers. Photoreceptor layer thickness was defined as the distance between the inner nuclear layer and RPE while RPE–BM was defined as between the RPE and Bruch’s membrane (Fig 1). Retinal sublayers for the 4 parafoveal subfields for PRL and RPE–BM were analyzed individually and as an average of all subfields (Fig 1). Standard criteria for quality assessment of OCT in UKBB have been previously described.^23^^,^^24^ We excluded the poorest 20% of images based on specific image quality metadata, generated by Topcon Advanced Boundary Segmentation for each OCT volume.

### Exposure Variables

The primary exposure variable was self-reported periodontitis. Individuals reporting painful gums or loose teeth were considered as having very severe periodontitis based on the findings of previous validity studies.^25^, ^26^, ^27^ We excluded individuals reporting denture wear as they were unable to report the exposure (loose teeth) and the origin of their denture wear was not recorded. We also excluded individuals with bleeding gums as this symptom is common among the general population (> 50% in a recent UK-based survey^28^), and previous literature suggests poor diagnostic accuracy for periodontitis with this question.^26^ We additionally performed a sensitivity analysis including, as cases, only individuals reporting loose teeth, as this has previously been shown to have the highest sensitivity and specificity for severe periodontitis among the items in the questionnaire.^26^ As controls, we excluded those with dentures, gingival bleeding, mouth ulcers, and toothache.

Secondary exposure variables were defined a priori and included age, sex, ethnicity, socioeconomic status, diabetes mellitus, hypertension, alcohol drinker status, smoking status, refractive error, and previous cataract surgery. Socioeconomic status was measured using the Townsend deprivation scores, a relative measure of material deprivation derived from 4 areas: unemployment, nonhome ownership, noncar ownership, and household overcrowding.^29^ Hypertension and diabetes mellitus were self-reported by the participant through touchscreen questionnaire. For hypertension, all those who reported having either hypertension or essential hypertension were included. For diabetes mellitus, all those reporting diabetes, type 1 or type 2 diabetes mellitus, were categorized into a binary variable of diabetic/nondiabetic. Smoking status was also reported by participants as never, previous, or current. The few who preferred not to answer this question at the initial visit were excluded (499 461/501 518 [99.6%] complete). Alcohol drinker status was self-reported as “never,” “previous,” or “current,” and was available for 500 757 of 501 512 participants (99.8%). Refractive error, as measured using the spherical equivalent on autorefraction, is strongly associated with retinal thicknesses on OCT,^24^ and was included as an adjustment variable. Given that refractive error will be influenced by previous cataract surgery, we additionally adjusted for this using the self-reported data in UKBB at the eye level.

### Data Analysis

Distribution of data was visualized using quantile–quantile plots and assessed statistically with the Anderson–Darling test; homogeneity of variance was tested using Levene’s test. Continuous variables were summarized using mean ± standard deviation and categorical variables through percentages. Comparison of PRL and RPE–BM thickness between groups was assessed using the independent samples *t* test (where data from both eyes were available, we averaged the measurement from both for unadjusted analyses). Chi-square testing was used to assess the proportional association between periodontal disease and categorical secondary exposure variables. For adjusted analyses, we fitted linear mixed effects regression models using maximum likelihood estimation with a random effect on the intercept. Models were adjusted for age, sex, ethnicity, socioeconomic status, diabetes mellitus, hypertension, alcohol drinker status, smoking status, refractive error, and previous cataract surgery. Degrees of freedom for multilevel modeling were estimated using Satterthwaite’s approximation.^30^ We assessed for interactions between very severe periodontitis and age, smoking status, and diabetes mellitus by comparing models with and without an interaction term using the likelihood ratio test (LRT)/Wilks test to compare model fit.^31^ The level of statistical significance was set at *P* < 0.05. All analyses were conducted in R version 4.1.0 (R Core Team, 2021. R Foundation for Statistical Computing) and used the lme4 and lmertest packages.^32^, ^33^, ^34^

Ethics committee approval was obtained for UKBB (ref: 06/MRE08/75); specific approval was obtained for this project (application ID: 2112). This study adhered to the ethical standards outlined in the Declaration of Helsinki.

## Results

**Figure 2:**
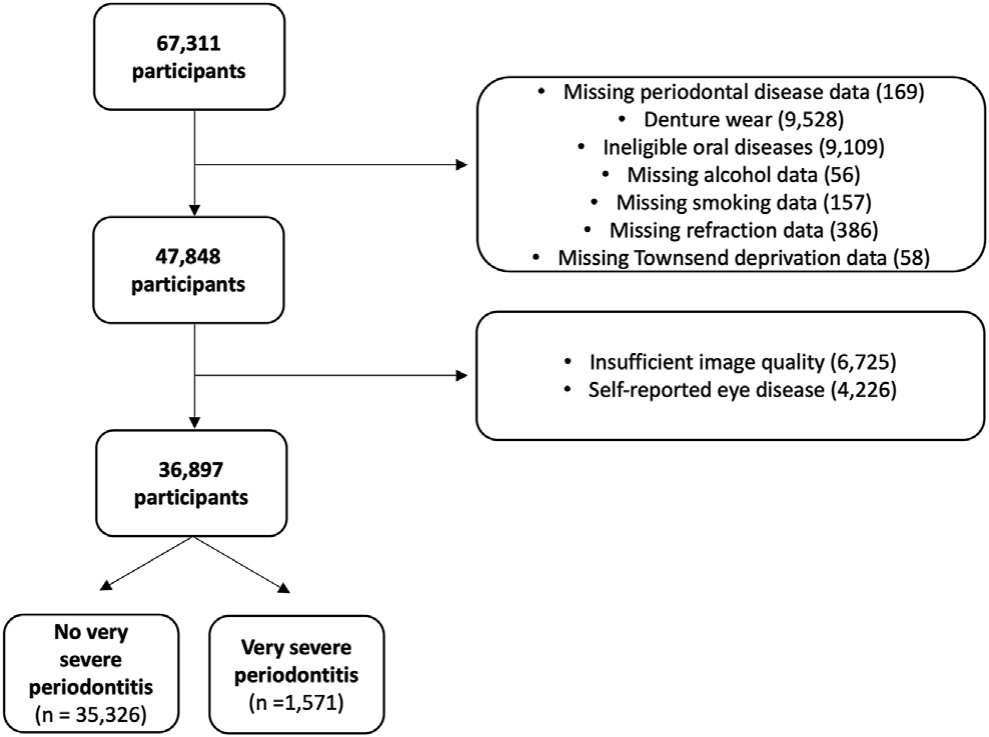
Flow chart of included participants.

From an initial cohort of 67 311 participants who underwent retinal imaging at the initial visit, there were 1571 individuals (2748 eyes) with very severe periodontitis and 35 326 unaffected individuals (62 221 eyes) included in the analysis (prevalence: 4.3%, Fig 2). Individuals with very severe periodontitis were older (56.6 ± 7.8 years vs. 55.6 ± 8.1 years, *P* < 0.001) and lived in areas of greater socioeconomic deprivation (Townsend score: −0.43 ± 3.2 vs. −1.22 ± 2.9, *P* <0.001). They were also more likely to be current smokers (18.8% vs. 8.9%, *P* < 0.001) and have hypertension (26.2% vs. 22.7%, *P* < 0.001) and diabetes mellitus (5.3% vs. 3.3%, *P* < 0.001). On average, those with very severe periodontitis were hyperopic (0.05 ± 2.27 diopters) while those unaffected were myopic (−0.29 ± 2.40 diopters, *P* < 0.001). On unadjusted analysis, individuals with very severe periodontitis had thinner PRL (severe periodontitis: 164.3 ± 9.0 μm vs. unaffected: 165.2 ± 8.8 μm, *P* < 0.001) but did not differ significantly in RPE–BM thickness (severe periodontitis: 23.0 ± 2.1 μm vs. unaffected: 22.9 ± 2.5 μm, *P* = 0.40, Table 1).

**Table 1:** Baseline Characteristics of the Cohort

Characteristic∗	No Very Severe Periodontitis (n = 35 326)	Very Severe Periodontitis (n = 1571)	*P* Value
Age, mean ± SD (median, IQR)			
Yrs	55.6 **±** 8.1 (56, 49.5–62.5)	56.6 **±** 7.8 (58, 52–64)	**< 0.001**
Sex n (%)			
Female	19 167 (54.3)	845 (53.8)	0.73
Male	16 159 (45.7)	726 (46.2)	
Ethnicity n (%)			
Asian (South)	923 (2.6)	90 (5.7)	**< 0.001**
Black	908 (2.6)	68 (4.3)	
Other	995 (2.8)	86 (5.5)	
White	32 500 (92.0)	1327 (84.5)	
Socioeconomic status, mean ± SD (median, IQR)			
Townsend score	−1.22 **±** 2.9 (−1.84, –3.89 to 0.21)	−0.43 **±** 3.2 (−1.00, −3.45 to 1.45)	**< 0.001**
Diabetes mellitus n (%)			
Absent	34 151 (96.7)	1487 (94.7)	**< 0.001**
Present	1175 (3.3)	84 (5.3)	
Hypertension n (%)			
Absent	27 306 (77.3)	1159 (73.8)	**0.001**
Present	8020 (22.7)	412 (26.2)	
Alcohol drinker status n (%)			
Never	1469 (4.2)	110 (7.0)	**< 0.001**
Previous	1083 (3.1)	77 (4.9)	
Current	32 774 (92.8)	1384 (88.1)	
Smoking status n (%)			
Never	20 553 (58.2)	729 (46.5)	**< 0.001**
Previous	11 649 (33.0)	546 (34.8)	
Current	3124 (8.9)	296 (18.8)	
Refractive error, mean ± SD			
Diopters	−0.29 **±** 2.40	0.05 **±** 2.27	**< 0.001**
Retinal layer thicknesses, mean ± SD			
PRL (μm)	165.2 ± 8.8	164.3 ± 9.0	**< 0.001**
RPE–BM (μm)	22.9 **±** 2.5	23.0 **±** 2.1	0.40

Figures in bold were considered statistically significant.

IQR = interquartile range; PRL = photoreceptor layer; RPE–BM = retinal pigment epithelium–basement membrane; SD = standard deviation.

∗ Where data on both eyes were available, the values for retinal layer thicknesses and refractive error were averaged. This included 35 326 participants with 62 221 eyes as controls and 1571 participants with 62 221 eyes as cases.

**Figure 3:**
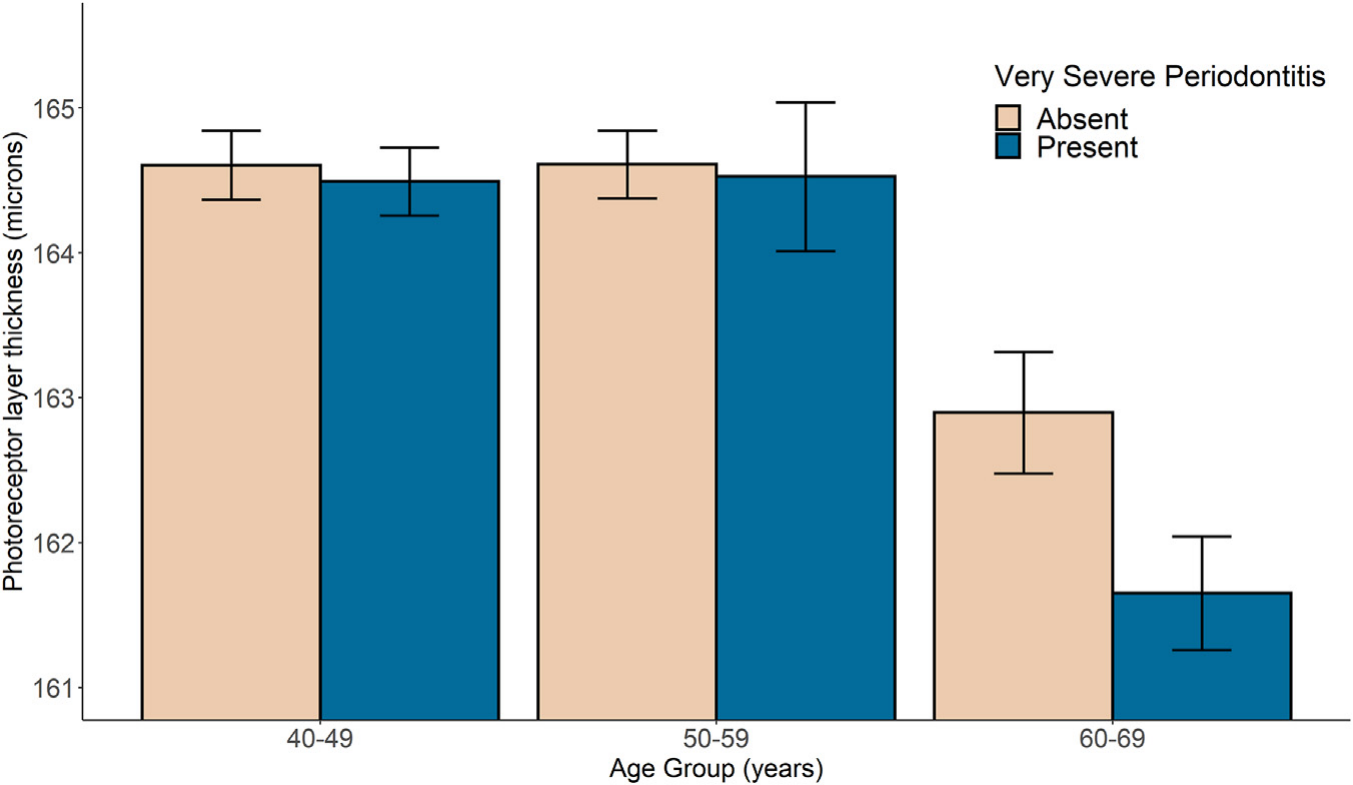
Difference in photoreceptor layer thickness between those with and without very severe periodontitis grouped by age. A significant difference in thickness of the sublayer was only seen among those aged 60 to 69 years. Error bars indicate 95% confidence intervals.

Adjusting for all confounders, very severe periodontitis was associated with thinner PRL (−0.55 μm; 95% confidence interval [CI], −0.97 to −0.12; *P* = 0.013). Photoreceptor layer thickness difference was greatest in the superior parafoveal segment (−0.70 μm; 95% CI, −1.14 to −0.26; *P* = 0.002; Table S2, available at www.ophthalmologyscience.org). Thinner PRL was also associated with older age, non-White ethnicity, diabetes mellitus, hypertension, and current smoking (Table 3). There was no significant difference in RPE−BM layer thickness between unaffected individuals and those with very severe periodontitis (0.00 μm; 95% CI, −0.12 to 0.13, *P* = 0.97). The RPE–BM was thicker among older individuals (0.22 μm per decile; 95% CI, 0.19–0.25; *P* < 0.001), men (0.32 μm; 95% CI, 0.27–0.37; *P* < 0.001), and those self-reporting Black (1.57 μm; 95% CI, 1.41–1.73; *P* < 0.001) or South Asian (0.31 μm; 95% CI, 0.14–0.47; *P* < 0.001) ethnicity. There was no evidence of interaction between current smoking (LRT, *P* = 0.26) or diabetes mellitus (LRT, *P* = 0.56) and very severe periodontitis on PRL thickness. However, there was evidence of interaction between age and very severe periodontitis for PRL thickness (LRT, *P* < 0.001). When stratifying individuals by age, we found PRL was thinner among those aged 60 to 69 years (−1.19 μm; 95% CI, −1.85 to −0.53; *P* < 0.001), but not those aged 40 to 49 years or 50 to 59 years (Fig 3, Table 4). On the sensitivity analysis, similar direction but more extreme effect estimates were found, with affected individuals having a −0.90 μm (95% CI, −1.49 to −0.30) thinner PSL. Those with very severe periodontitis also had a thicker RPE−BM layer (0.89 μm; 95% CI, 0.33–1.46; *P* = 0.002; Table S5, available at www.ophthalmologyscience.org).

**Table 3:** Thickness Differences of the Photoreceptor and RPE–BM Layers Estimated through Multivariable Linear Mixed Effects Models

Variable	PRL (μm)		RPE–BM (μm)	
	Thickness Difference (95% CI)	*P* Value	Thickness Difference (95% CI)	*P* Value
Very severe periodontitis				
Absent	Reference		Reference	
Present	−0.55 (−0.97 to −0.12)	**0.013**	0.00 (−0.12 to 0.13)	0.97
Age				
Per decile	−0.99 (−1.11 to −0.88)	**< 0.001**	0.22 (0.19–0.25)	**< 0.001**
Sex				
Female	Reference		Reference	
Male	1.91 (1.74–2.09)	**< 0.001**	0.32 (0.27–0.37)	**< 0.001**
Ethnicity				
White	Reference		Reference	
Asian (South)	−3.94 (−4.49 to −3.39)	**< 0.001**	0.31 (0.14–0.47)	**< 0.001**
Black	−5.85 (−6.41 to −5.29)	**< 0.001**	1.57 (1.41–1.73)	**< 0.001**
Other	−2.29 (−2.81 to −1.77)	**< 0.001**	0.47 (0.32–0.62)	**< 0.001**
Socioeconomic status				
Per SD increase	−0.28 (−0.37 to −0.19)	**< 0.001**	0.00 (−0.03 to 0.02)	0.92
Diabetes mellitus				
Absent	Reference		Reference	
Present	−1.55 (−2.03 to −1.06)	**< 0.001**	0.05 (−0.10 to 0.19)	0.52
Hypertension				
Absent	Reference		Reference	
Present	−0.82 (−1.04 to −0.61)	**< 0.001**	−0.02 (−0.08 to 0.05)	0.62
Alcohol drinker status				
Never	Reference		Reference	
Previous	0.65 (0.00–1.30)	0.05	0.00 (−0.19 to 0.19)	0.97
Current	1.09 (0.64–1.53)	**< 0.001**	−0.02 (−0.15 to 0.11)	0.81
Smoking status				
Never	Reference		Reference	
Previous	0.07 (−0.13 to 0.26)	0.50	0.01 (−0.04 to 0.07)	0.60
Current	−0.73 (−1.04 to −0.42)	**< 0.001**	−0.12 (−0.21 to −0.03)	**0.008**
Refractive error				
Per diopter	1.69 (1.62–1.76)	**< 0.001**	−0.12 (−0.14 to −0.09)	**< 0.001**
Previous cataract surgery				
Absent	Reference		Reference	
Present	0.44 (−0.95 to 1.82)	0.54	0.21 (−0.34 to 0.77)	0.45

Figures in bold were considered statistically significant.

CI = confidence interval; PRL = photoreceptor layer; RPE–BM = retinal pigment epithelium–basement membrane; SD = standard deviation.

**Table 4:** Thickness Difference Estimates Stratified by Age Groups for the PRL

PRL Thickness	40 to 49 Age Group (n = 9855)		50 to 59 Age Group (n = 12 590)		60 to 69 Age Group (n = 14 452)	
	Thickness Difference (95% CI)	*P* Value	Thickness Difference (95% CI)	*P* Value	Thickness Difference (95% CI)	*P* Value
Very severe periodontitis						
Absent	Reference		Reference		Reference	
Present	−0.27 (−1.19 to 0.64)	0.56	−0.02 (−0.73 to 0.69)	0.95	−1.19 (−1.85 to −0.53)	< 0.001
Age						
Per decile	0.78 (0.18–1.37)	**0.010**	−1.11 (−1.55 to −0.52)	**< 0.001**	−2.30 (−2.81 to −1.79)	**< 0.001**
Sex						
Female	Reference		Reference		Reference	
Male	2.66 (2.32–2.99)	**<0.001**	1.56 (1.26– to 1.86)	**< 0.001**	1.81 (1.53–2.09)	**< 0.001**
Ethnicity						
White	Reference		Reference		Reference	
Asian (South)	−3.41 (−4.26 to −2.56)	**< 0.001**	−4.22 (−5.17 to −3.28)	**< 0.001**	−4.20 (−5.32 to −3.07)	**< 0.001**
Black	−6.59 (−7.37 to −5.81)	**< 0.001**	−5.50 (−6.43 to −4.57)	**< 0.001**	−4.36 (−5.93 to −2.80)	**< 0.001**
Other	−2.33 (−3.13 to −1.53)	**< 0.001**	−2.92 (−3.77 to −2.07)	**< 0.001**	−1.15 (−2.27 to −0.02)	**0.046**
Socioeconomic status						
Per SD increase	−0.13 (−0.31 to 0.04)	0.13	−0.35 (−0.50 to −0.20)	**< 0.001**	−0.28 (−0.42 to −0.14)	**< 0.001**
Diabetes mellitus						
Absent	Reference		Reference		Reference	
Present	−1.52 (−2.84 to −0.21)	**0.023**	−2.24 (−3.12 to −1.37)	**< 0.001**	−1.14 (−1.79 to −0.49)	**< 0.001**
Hypertension						
Absent	Reference		Reference		Reference	
Present	−0.47 (−1.02 to 0.07)	0.09	−0.61 (−0.98 to −0.24)	**0.001**	−1.04 (−1.34 to −0.74)	**< 0.001**
Alcohol drinker status						
Never	Reference		Reference		Reference	
Previous	0.24 (−0.96 to 1.44)	0.69	0.73 (−0.39 to 1.85)	0.20	0.66 (−0.40 to 1.72)	0.22
Current	1.03 (0.20–1.85)	**0.015**	1.03 (0.23–1.82)	**0.012**	1.03 (0.33–1.74)	**0.004**
Smoking status						
Never	Reference		Reference		Reference	
Previous	0.06 (−0.33 to 0.45)	0.77	0.18 (−0.15 to 0.51)	0.29	0.05 (−0.24 to 0.35)	0.73
Current	−0.36 (−0.87 to 0.15)	0.16	−0.60 (−1.12 to −0.07)	**0.026**	−1.44 (−2.02 to −0.85)	**< 0.001**
Refractive error						
Per diopter	1.80 (1.67–1.92)	**< 0.001**	1.69 (1.58–1.80)	**< 0.001**	1.61 (1.50–1.72)	**< 0.001**
Previous cataract surgery						
Absent	Reference		Reference		Reference	
Present	0.57 (−7.57 to 8.71)	0.89	−1.33 (−5.43 to 2.77)	0.53	0.83 (−0.72 to 2.37)	0.29

A significant association was only seen for the group aged 60 to 69 years. Figures in bold were considered statistically significant.

CI = confidence interval; PRL = photoreceptor layer; SD = standard deviation.

## Discussion

In this analysis of 36 948 participants in the UKBB who underwent retinal imaging and denied any eye disease, we found individuals with very severe periodontitis had thinner PRL. Thinner PRL was most marked in the superior parafoveal region and was only noted among those aged 60 to 69 years. Our report, the first to examine retinal OCT in periodontal disease, suggests individuals with very severe periodontitis have outer retinal features consistent with emerging AMD and support further investigation into the role of periodontal disease and oral hygiene in AMD incidence.

Our adjusted analysis showed that the PRL of individuals with very severe periodontitis was, on average, 0.55 μm thinner than that of controls, but this was driven predominantly by differences in the 60 to 69 year age group (−1.19 μm, 95% CI, −1.85 to −0.53). For context, this difference in PRL thickness was analogous to approximately 5 years of age and slightly smaller than the estimate for current smoking (−1.44 μm). The replication of similar directions and sizes of effect between PRL thickness and age, sex, ethnicity, hypertension, and current smoking reported in previous literature lends validity to our analyses.^35^, ^36^, ^37^ Although thinner PRL was originally noted as a feature of late AMD, its presence in early disease is increasingly recognized. The German AugUR study showed that, compared with normal eyes, individuals with moderate early AMD had a 1.7-μm thinner PRL within the central fovea subfield, whereas differences in the parafoveal subfield were more subtle (Figure 2 within their report^20^). Individuals with early AMD also have significantly thinner outer nuclear layers compared with controls,^20^ and recent evidence has suggested that PRL thinning may be the earliest manifestation of emerging AMD.^21^ Even among those with normal eyes, Zekavat et al^21^ showed that for each standard deviation decrease in PRL thickness, the incident risk of AMD diagnosis was increased by 14%; however, it should be noted that they did not include the outer nuclear layer in their definition of the PRL. Although there has been no previous report examining retinal OCT in individuals with periodontitis, our findings concord with epidemiological reports that have highlighted an association between periodontitis and AMD, as measured on CFP, in younger individuals. Participants in the United States-based National Health and Nutrition Examination Survey, who were aged ≤ 60 years and had periodontal disease, were more likely to have any form of AMD.^14^ This was echoed in a similar report in the Korean National Health and Nutrition Examination Survey where those aged ≤ 62 years with severe periodontal disease had 61% greater odds of having AMD.^15^ Although strengths of both of these reports include robust standardized definitions for AMD (CFP labeled by retinal specialists with expertise in AMD grading) and periodontal disease (through oral health examination by trained dentists according to World Health Organization criteria), there is considerable interobserver variability in CFP-based diagnosis of AMD. For example, in the Age-Related Eye Disease Study, whereas agreement was good for identifying the presence of advanced AMD (kappa: 0.88), it was more modest when considering features of earlier disease, such as depigmentation in the central zone (weighted kappa: 0.49).^38^ OCT imaging is more sensitive for detecting features of early AMD,^39^ and the use of a reproducible and quantifiable biomarker in our report not only mitigates the potential bias imparted by human-based dichotomization of a disease spectrum into presence or absence but also allows a deeper exploration into the early stages of AMD.

We did not find an association between very severe periodontitis and RPE–BM thickness in our primary analysis. The mean RPE–BM thickness of control participants (22.9 μm) was similar to that reported in normal eyes elsewhere,^40^^,^^41^ and apart from age, sex, ethnicity, and refractive error, we did not find any significant association between RPE−BM thickness and the confounders defined a priori. Similar findings were seen in the population-based Beijing Eye Study. Although age and hypertension were associated with thicker RPE–BM thickness on unadjusted analysis, they found no such link with alcohol consumption or diabetes mellitus.^41^ Several reports have noted an increase in RPE–BM thickness with age^42^ and in AMD^40^ owing to loss of melanin granules, calcification, and the accumulation of lipid and residual bodies.^43^ However, the sequence of outer retinal layer-specific changes remains unclear (e.g., whether photoreceptor thinning predates RPE–BM thickening or vice-versa). Although beyond the scope of our cross-sectional analysis, our findings do align with the conclusion of Zekavat et al^21^ that PRL thinning may predate RPE−BM thickening, at least in individuals with very severe periodontitis. To explore the potential causal relationship here, future work should longitudinally explore rates of PRL decline and RPE−BM thickening respectively in those with very severe periodontitis.

Periodontitis is associated with heightened systemic inflammation and addressing it through dental treatments leads to a reduction in inflammatory markers.^44^, ^45^, ^46^, ^47^, ^48^, ^49^ Given the role of systemic inflammation in the pathophysiology of AMD,^50^, ^51^, ^52^ it seems plausible that the association between periodontal disease and the outer retinal differences we describe are mediated via this pathway and anti-inflammatory measures may have beneficial effects on outer retinal health. Indeed, lifestyle measures which reduce systemic inflammatory burden, such as smoking cessation and vitamin supplementation, reduce the progression of dry AMD. Current smokers develop neovascular AMD 4.4 years younger than ex-smokers, which suggests cessation may have some benefit even when disease is established.^53^ Ultimately, future work should consider the impact of enhanced oral hygiene in individuals with periodontal disease on AMD onset, progression, and transformation from dry disease to choroidal neovascularization. Whether such measures could also alter the response to intravitreal therapy is also credible; sustained complement activation and inflammation are posited to underlie resistance to anti-VEGF treatment,^54^ and intravitreal steroid has demonstrated efficacy in reducing retinal thickness and intraretinal fluid in neovascular AMD.^55^^,^^56^

Strengths of our report include a large population-based cohort, rich deeply phenotyping data permitting the adjustment for probable confounders, and standardized retinal imaging acquisition with reproducible image segmentation. However, there are also limitations. We defined very severe periodontitis as those self-reporting loose teeth and painful gums based on previously published work on the validity of self-reporting for periodontitis. Although self-reporting loose teeth has high pooled specificity for periodontitis (moderate: 94.7, severe: 91.9), the pooled sensitivity is low, ranging from 28.3 for moderate disease to 54.9 for severe disease.^26^ Thus, although individuals with self-reported loose teeth are likely to have very severe periodontitis, it is likely that some controls may also have periodontitis, suggesting a dilution of any measure of effect. Indeed, the prevalence of very severe periodontitis within this cohort was at the lower end of estimates across the UK.^4^ Other prospective cohort studies have used oral health examination by licensed dentists, or even incorporated dental radiographs for the case definition,^13^^,^^14^ and this may be considered for future work. Similarly, we did not have data on the duration of periodontitis. The UKBB touchscreen questionnaire asks about relevant symptoms within the last year but it is likely that disease duration was heterogeneous among our cases. This report should also be considered in the context of the potential selection bias of UKBB. As a population-based cohort of healthy volunteers with an exceptionally low response rate (∼ 6%), there have been some concerns over extrapolating the findings derived from UKBB participants. Compared with the general population, participants in UKBB are less likely to engage in harmful health behaviors and experience less socioeconomic deprivation.^57^ The UKBB participants are also predominantly of White ethnicity, suggesting our findings should be interpreted with caution in other ethnic groups. However, risk factor associations estimated from UKBB have been found to be generalizable when pooling data from other nationally sampled cohort studies within England.^58^

In conclusion, individuals with severe periodontitis and no known eye disease have measurable differences in the thickness of the PRL. Although longitudinal analyses are needed to further confirm, the directions of effect are consistent with those seen in emerging AMD and remain significant despite adjustment for known confounding factors, including current smoking. Recommendations on oral hygiene may hold additional relevance for people at risk of degenerative retinal disease.
