# Supplementary material for: Periodontitis and Outer Retinal Thickness: a Cross-Sectional Analysis of the United Kingdom Biobank Cohort
Source: Ophthalmol Sci. 2024 Jan 20;4(4):100472. doi: 10.1016/j.xops.2024.100472 (PMC10973663; doi:10.1016/j.xops.2024.100472)
Supplement: Table S2 [file mmc1.pdf]

Characteristic		Layer thickness difference (95% CI)	*p*-value
**PRL (μm)**	**All subfields**	-0.55 (-0.97, -0.12)	**0.013**
	**Inner superior**	-0.70 (-1.14, -0.26)	**0.002**
	**Inner nasal**	-0.54 (-0.99, -.0.08)	**0.021**
	**Inner temporal**	-0.59 (-1.03, -0.15)	**0.009**
	**Inner inferior**	-0.36 (-0.80, 0.09)	0.12
**RPE-BM (μm)**	**All subfields**	0.00 (-0.12, 0.13)	0.97
	**Inner superior**	-0.03 (-0.16, 0.10)	0.68
	**Inner nasal**	-0.04 (-0.19, 0.10)	0.54
	**Inner temporal**	0.06 (-0.08, 0.20)	0.42
	**Inner inferior**	0.00 (-0.16, 0.15)	0.98

Supplementary Table 2: Thickness difference estimates for the average and each of the parafoveal fields.

CI: confidence interval, IMD: index of multiple deprivation, PRL: photoreceptor layer, RPE-BM: retinal pigment epithelium-basement membrane, μm: microns
