# Supplementary material for: Periodontitis and Outer Retinal Thickness: a Cross-Sectional Analysis of the United Kingdom Biobank Cohort
Source: Ophthalmol Sci. 2024 Jan 20;4(4):100472. doi: 10.1016/j.xops.2024.100472 (PMC10973663; doi:10.1016/j.xops.2024.100472)
Supplement: Table S5 [file mmc2.pdf]

| Variable | | PRL (µm) | | RPE-BM (µm) | |
|---|---|---|---|---|---|
| | | **Thickness difference (95% CI)** | ***p*-value** | **Thickness difference (95% CI)** | ***p*-value** |
| **Very severe periodontitis** | Absent | **Reference** | | **Reference** | |
| | Present | -0.90 (-1.49, -0.30) | **0.003** | 0.89 (0.33, 1.46) | **0.002** |
| **Age** | Per decile | -1.07 (-1.21, -0.92) | **<0.001** | 0.28 (0.14, 0.42) | **<0.001** |
| **Sex** | Female | **Reference** | | **Reference** | |
| | Male | 2.03 (1.81, 2.25) | **<0.001** | 0.04 (-0.16, 0.25) | 0.67 |
| **Ethnicity** | White | **Reference** | | **Reference** | |
| | Asian (South) | -3.93 (-4.63, -3.24) | **<0.001** | 0.70 (0.04, 1.36) | **0.039** |
| | Black | -5.62 (-6.32, -4.92) | **<0.001** | 1.28 (0.61, 1.94) | **<0.001** |
| | Other | -2.10 (-2.75, -1.46) | **<0.001** | 0.54 (-0.07, 1.15) | 0.08 |
| **Socioeconomic status** | Per SD increase | -0.42 (-0.53, -0.30) | **<0.001** | 0.05 (-0.05, 0.16) | 0.33 |
| **Diabetes mellitus** | Absent | **Reference** | | **Reference** | |
| | Present | -1.47 (-2.07, -0.87) | **<0.001** | 0.25 (-0.31, 0.82) | 0.38 |
| **Hypertension** | Absent | **Reference** | | **Reference** | |
| | Present | -0.98 (-1.25, -0.71) | **<0.001** | 0.07 (-0.18, 0.33) | 0.57 |
| **Alcohol drinker status** | Never | **Reference** | | **Reference** | |
| | Previous | 0.56 (-0.26, 1.39) | 0.18 | -0.20 (-0.98, 0.58) | 0.62 |
| | Current | 0.80 (0.24, 1.37) | **0.005** | -0.12 (-0.66, 0.41) | 0.65 |
| **Smoking status** | Never | **Reference** | | **Reference** | |
| | Previous | 0.13 (-0.11, 0.37) | 0.29 | -0.09 (-0.32, 0.14) | 0.44 |
| | Current | -0.65 (-1.04, -0.27) | **<0.001** | -0.10 (-0.26, 0.47) | 0.59 |
| **Refractive error** | Per dioptre | 1.77 (1.67, 1.86) | **<0.001** | 0.03 (-0.07, 0.13) | 0.55 |

Supplementary Table 5: Results of the sensitivity analysis with thickness differences of the photoreceptor segment and retinal pigment epithelium-basement membrane layers estimated through multivariable linear mixed effects models.

CI: confidence interval, IMD: index of multiple deprivation, PRL: photoreceptor layer, RPE-BM: retinal pigment epithelium-basement membrane, SD: standard deviation.
